# Effects of the probiotic formulation SLAB51 in *in vitro* and *in vivo* Parkinson’s disease models

**DOI:** 10.18632/aging.102927

**Published:** 2020-03-09

**Authors:** Vanessa Castelli, Michele d’Angelo, Francesca Lombardi, Margherita Alfonsetti, Andrea Antonosante, Mariano Catanesi, Elisabetta Benedetti, Paola Palumbo, Maria Grazia Cifone, Antonio Giordano, Giovambattista Desideri, Annamaria Cimini

**Affiliations:** 1 Department of Life, Health and Environmental Sciences, University of L’Aquila, 67100 L’Aquila, Italy; 2 Department of Medical Biotechnology, University of Siena, Siena, Italy; 3 Sbarro Institute for Cancer Research and Molecular Medicine and Center for Biotechnology, Temple University, Philadelphia, PA 19122, USA

**Keywords:** probiotics, Parkinson's disease, BDNF, tyrosine hydroxylase, 6-OHDA

## Abstract

Parkinson is a common neurodegenerative disorder, characterized by motor and non-motor symptoms, including abnormalities in the gut function, which may appear before the motor sign. To date, there are treatments that can help relieve Parkinson’ disease (PD)-associated symptoms, but there is no cure to control the onset and progression of this disorder. Altered components of the gut could represent a key role in gut-brain axis, which is a bidirectional system between the central nervous system and the enteric nervous system. Diet can alter the microbiota composition, affecting gut-brain axis function. Gut microbiome restoration through selected probiotics’ administration has been reported. In this study, we investigated the effects of the novel formulation SLAB51 in PD. Our findings indicate that this probiotic formulation can counteract the detrimental effect of 6-OHDA *in vitro* and *in vivo* models of PD. The results suggest that SLAB51 can be a promising candidate for the prevention or as coadjuvant treatment of PD.

## INTRODUCTION

Neurodegenerative disease etiology is still unclear, but different contributing factors, such as lifestyle and genetic factors are involved [1]. Parkinson is a common neurodegenerative disease, characterized by loss of dopaminergic neurons and α-synuclein intracellular accumulation, named Lewy bodies [2]. Numerous studies have indicated that the underlying mechanisms of Parkinson’s disease (PD) involve the inflammatory pathway and the oxidative stress, characterized by an imbalance between protective and detrimental function [3–5]. Moreover, in neurodegenerative diseases, including PD, reduced neurotrophic support has been reported [6, 7], such as brain derived neurotrophic factor (BDNF).

Neuroinflammation and oxidative stress trigger to α-synuclein aggregation, which, in turn, lead to a stronger release of proinflammatory cytokines and reactive oxygen species (ROS) [8, 9]. PD pathology is indicated to begin in the *substantia nigra* but could involve also the enteric nervous system, highlighting the interaction between the gut and Central Nervous System (CNS) [10–12]. Clinically, PD patients present motor symptoms, including tremor, rigidity, postural instability, and bradykinesia, accompanied by non-motor symptoms, such as depression, abnormalities in the gut function, pain, hyposmia, which may appear before the motor sign [13–15]. Altered components of the gut could represent a key role in gut-brain axis, which is a bidirectional system between the CNS and the enteric nervous system. Diet can alter the microbiota composition, affecting gut-brain axis function [12, 16]. PD patients’ intestinal tract is characterized by pro-inflammatory microbiota, which induce improved gut permeability, known as “leaky gut”, in which inflammatory mediators and bacteria pass the mucosa and invade the blood [17]. Moreover, the loss of enteric dopaminergic neurons induces higher expression of pro-inflammatory cytokines [18]. The intestinal tract of PD patients shows a different gut microbiota compared to healthy individuals, with reduced levels of *Prevotellaceae* and abundance of *Enterobacteriaceae* [12, 19, 20], this dysbiosis is more evident in the severe PD phenotype.

The gut microbiome restoration has been reported to counteract PD progression and this effect can be exerted by probiotics, prebiotics and symbiotic formulations, fecal microbiota transplantation or stem cell transplant [21–23].

Probiotic formulations may dampen the inflammation through cytokines production [24], and decrease the oxidative stress through a reduction in ROS [25]. This aspect is of high interest since PD progression is accelerated in presence of infections [26]. It has been demonstrated that the original probiotic formulation DSF is able to control the expression of different genes in the brain cortex of aging animals, dampening the inflammation and improving neuronal performances [27]. More recently, a novel probiotic formulation (SLAB51) was investigated in an Alzheimer’s disease (AD) mouse model, exhibiting attenuation of cognitive impairment, reduction in Aβ aggregates and brain injuries and partial restoration of altered neuronal proteolytic pathway [28]. The same research group indicated a strong oxidative reduction upon SLAB51 treatment in the AD mouse brain, though SIRT1-dependent mechanisms [29].

In this background, the aim of this study was to investigate the effects of SLAB51 formulation in PD. To this purpose, we first tested the formulation on a PD *in vitro* model and after obtained interesting data, the potential therapeutic of SLAB51 was examined *in vivo* through dopaminergic neurons analyses and behavioral tests.

## RESULTS

### In vitro

**Figure 1:**
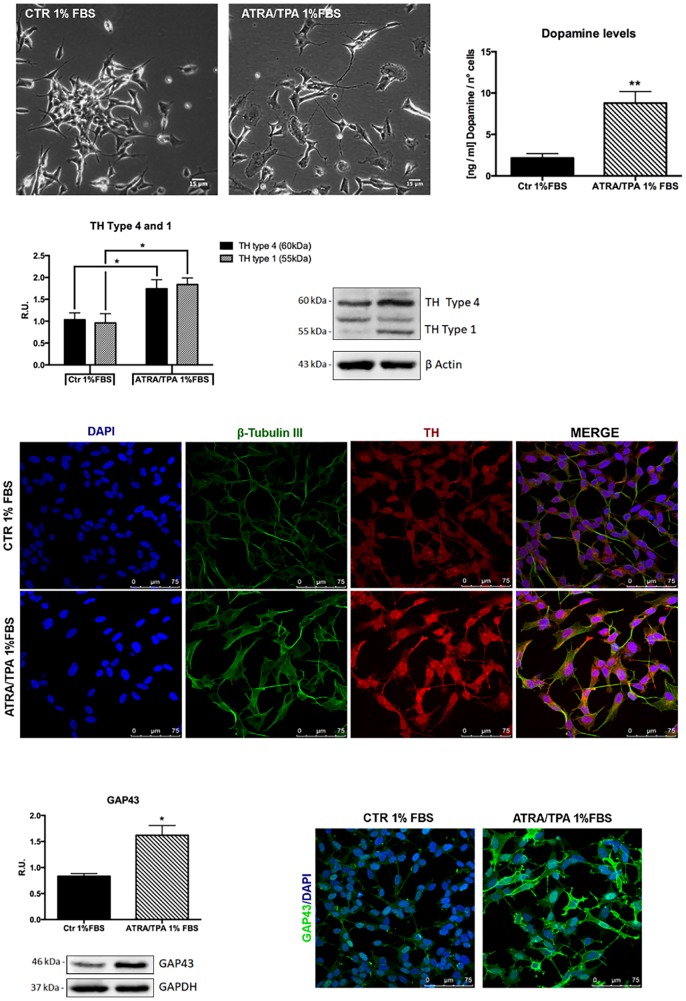
**Dopaminergic phenotype of SH-SY5Y neuroblastoma cells.** Contrast phase microscopy of differentiated with ATRA/TPA and not differentiated SH-SY5Y cells and histogram showing dopamine production. Western blotting for TH. Immunofluorescence of β-tubulin III and TH. Western blotting and immunofluorescence for GAP43. Results are mean ± SE of 3 different experiments (n=3). *p< 0,05, **p< 0,005 vs. ATRA/TPA.

To investigate if the probiotic formulation SLAB51 contained neuroprotective components, an *in vitro* model of PD was developed, and neuroprotective and neuronal death pathways were analyzed. The induction of the dopaminergic phenotype of SH-SY5Y neuroblastoma cells, described in the Methods section, was analyzed by contrast phase microscopy, Tyrosine Hydroxylase (TH) expression, dopamine production and immunofluorescence (Figure 1). It is possible to observe that, upon retinoic acid (RA)/phorbol ester 12-O-tetradecanoylphorbol-13-acetate (TPA) treatment, cells displayed neuronal morphology, expressed higher levels of TH, produced dopamine and expressed neuronal markers, such as β-tubulin III and growth associated protein-43 (GAP-43). 6-hydroxydopamine (6-OHDA) concentration able to induce a significant decrease of cell viability was evaluated by MTS assay (not shown). On this basis, 35 μM 6-OHDA was chosen for the subsequent experiments, since a 50% mortality with this dosage was obtained.

**Figure 2:**
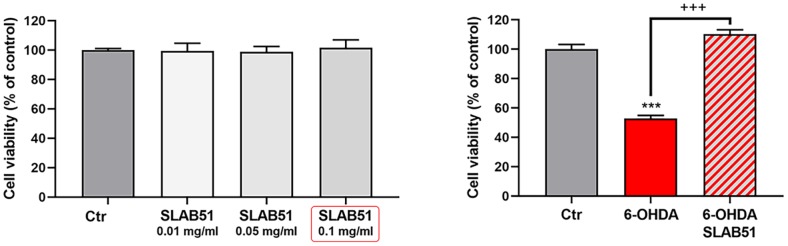
**MTS assay of cells treated with different concentration of SLAB51 (left).** MTS assay of cells treated with 35 μM 6-OHDA and 35 μM 6-OHDA and SLAB51 0.1mg/ml (right). Data are mean ± SE of three different experiments run in quadruplicate (n=3). *** p< 0.0005 vs Ctr; +++ p< 0.0005 vs 6-OHDA.

SLAB51 lysates do not shown toxic effect, as demonstrated by cell viability test on differentiated SH-SY5Y; thus, basing on these preliminary results, 0.1 mg/ml of extract was set as testing concentration for the subsequent experiments. In Figure 2, the MTS assay of cells treated with 35 μM 6-OHDA and SLAB51 is shown. As evident, 6-OHDA led to 50% mortality, while SLAB51 was able to counteract 6-OHDA-induced injury and to restore control conditions.

**Figure 3:**
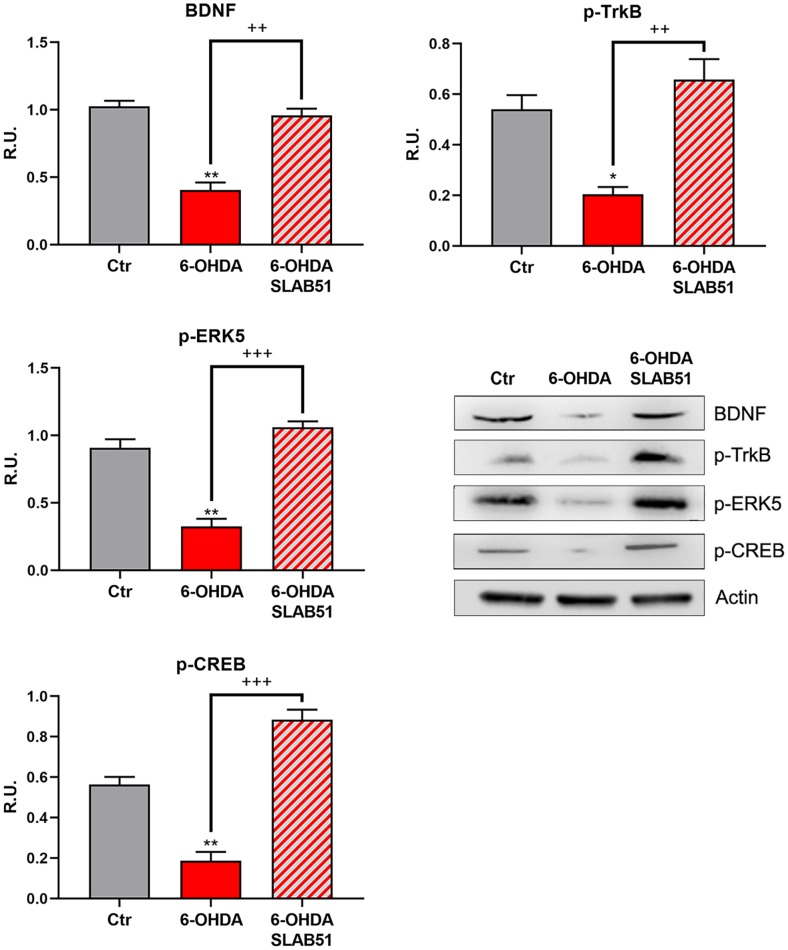
**WB and relative densitometric analysis for Ctr, 6-OHDA and 6-OHDA+SLAB51 for mBDNF, p-TrkB, p-ERK5, p-CREB.** Results are mean ± SE of 3 different experiments (n=3). *p< 0.05; ** p< 0.005 vs Ctr; ++ p< 0.005, +++ p< 0.0005 vs 6-OHDA. Representative WB figures are shown.

Since it is worth noting that BDNF pathway is involved in neuroprotection and neuronal survival, we first analyzed, by Western blotting, the protein levels of mature BDNF (mBDNF), phosphorylated tyrosine receptor kinase B (p-TrkB), phosphorylated cAMP response element-binding protein (p-CREB) and phosphorylated extracellular-signal-regulated kinase 5 (p-ERK5). In the presence of 6-OHDA, mBDNF and its specific receptor TrkB as well the survival kinase ERK5 were significantly decreased with respect to control neurons, while the presence of SLAB51 restored the control levels (Figure 3), suggesting a protective action exerted by the probiotic. Further, p-CREB, which is known to control mBDNF levels and the survival pathway phosphoinositide 3-kinase (PI3K)/ phosphorylated protein kinase B (p-Akt), was dramatically decreased upon 6-OHDA and restored at control levels by SLAB51 (Figure 3).

**Figure 4:**
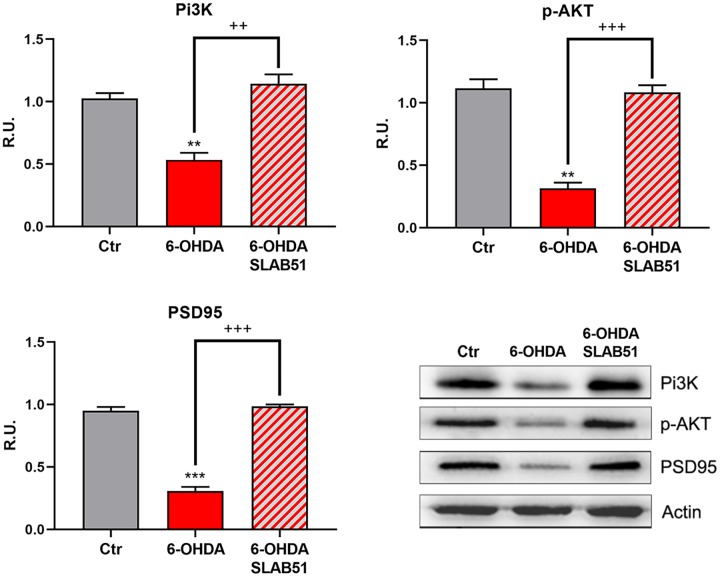
**WB and relative densitometric analysis for Ctr, 6-OHDA and 6-OHDA+SLAB51 for PI3K, p-Akt, and PSD95.** Results are mean ± SE of 3 different experiments (n=3). ** p< 0.005, ***p< 0.0005 vs Ctr; ++ p< 0.005, +++ p< 0.0005 vs 6-OHDA. Representative WB figures are shown.

Finally, PI3K/Akt pathway, which is involved in neuronal survival and CREB phosphorylation, as well as the postsynaptic density protein 95 (PSD95) appeared strongly downregulated by 6-OHDA, while the presence of SLAB51 counteracted this effect (Figure 4), thus suggesting that the lysate is able to ameliorate the neuronal synaptic plasticity as well the neuronal survival.

**Figure 5:**
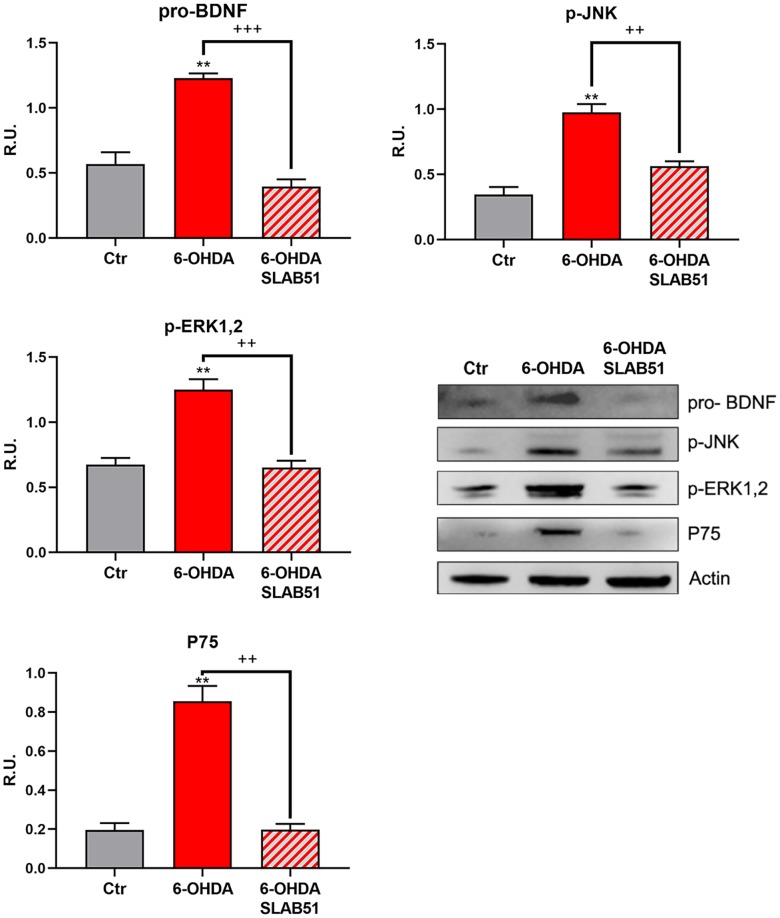
**WB and relative densitometric analysis for Ctr, 6-OHDA and 6-OHDA+SLAB51 for pro-BDNF, p-JNK, p-ERK1,2 and P75.** Results are mean ± SE of 3 different experiments (n=3). ** p< 0.005 vs Ctr; ++ p< 0.005, +++ p< 0.0005 vs 6-OHDA. Representative WB figures are shown.

The neuronal death pathway, comprising pro-BDNF, phosphorylated c-Jun N-terminal kinase (p-JNK), phosphorylated extracellular signal–regulated kinase (p-ERK1,2) and P75 was then analyzed. All these proteins were significantly increased by 6-OHDA, while the presence of SLAB51 restored the control conditions, thus confirming a neuroprotective role exerted by the probiotic (Figure 5).

**Figure 6:**
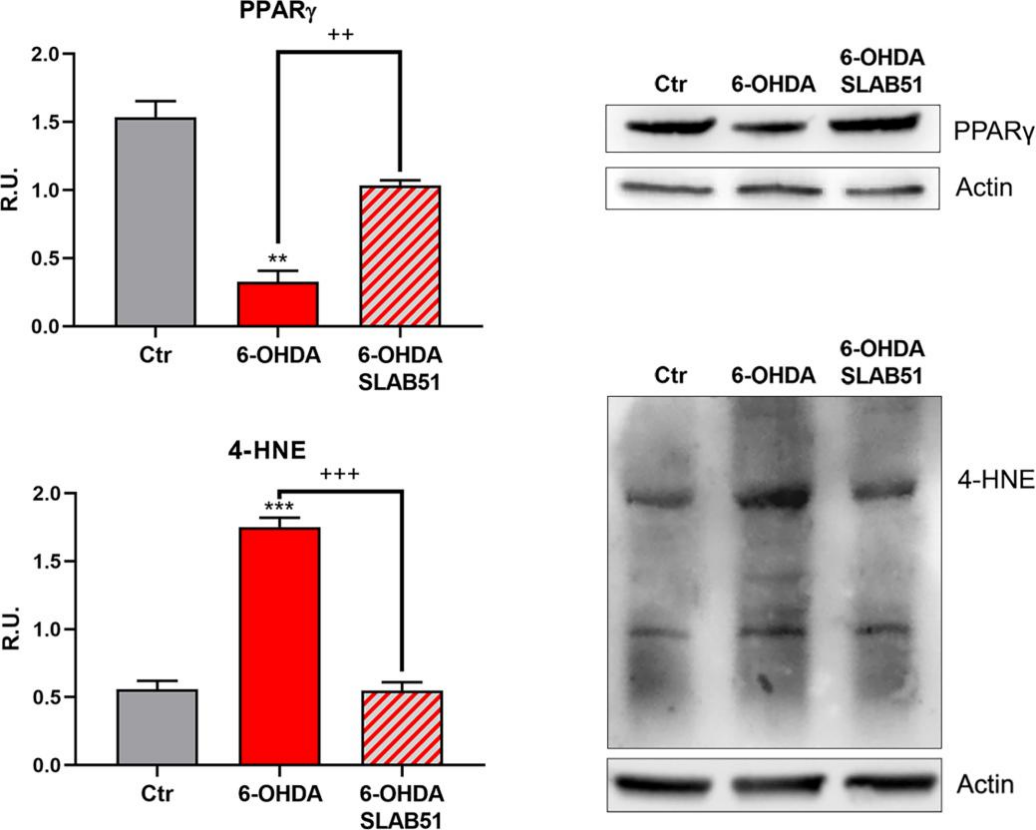
**WB and relative densitometric analysis for Ctr, 6-OHDA and 6-OHDA+SLAB51 for PPARγ and 4-HNE proteins adducts.** Results are mean ± SE of 3 different experiments (n=3). ** p< 0.005, ***p< 0.0005 vs Ctr; ++ p< 0.005, +++ p< 0.0005, vs 6-OHDA. Representative WB figures are shown.

Recent evidence reported the involvement of activated peroxisome proliferator activated receptor γ (PPARγ) in modulating BDNF levels in different pathologies, including PD [30]. Thus, on light of the results collected so far, we assayed PPARγ level through Western blotting analysis. Indeed, in 6-OHDA-treated cells a significant reduction of the transcription factor was apparent; while SLAB51 lysate increased PPARγ protein levels (Figure 6). Further, since it is known that a product of lipid peroxidation, 4-hydroxynonenal (4-HNE) is generally increased during oxidative stress, as occur in neurodegeneration processes, and to highlight a potential role of probiotics in counteracting 6-OHDA oxidative injury, 4-HNE protein adducts by Western blotting in *in vitro* samples were analyzed. It is possible to observe that the probiotic formulation significantly reduced the level of 4-HNE proteins adducts, thus suggesting a protective role of SLAB51 against oxidative damage (Figure 6). Once obtained the above promising results *in vitro*, in order to evaluate if this formulation was able to modulate neuroprotective pathways in a more complex model, we tested the probiotic in unilateral 6-OHDA-lesioned animal model.

### In vivo

**Figure 7:**
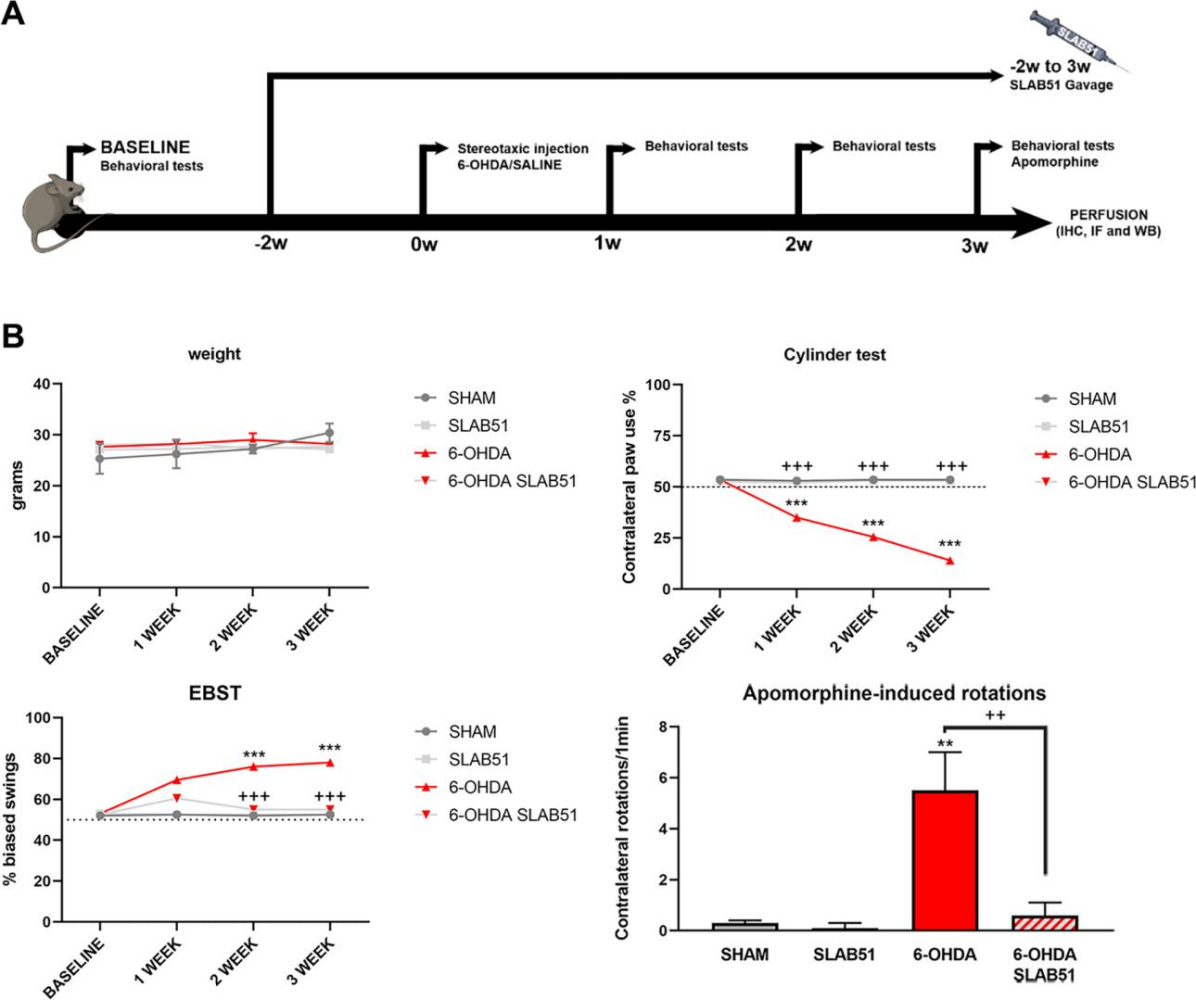
(**A**) Procedural timeline with specific timepoints. (**B**) Body weight and behavioral tests in SHAM, SLAB51, 6-OHDA and 6-OHDA+SLAB51 animals. ** p< 0.005, *** p< 0.0005 vs Ctr; ++ p< 0.005, +++ p< 0.0005 vs 6-OHDA.

To test the formulation *in vivo,* SLAB51 diluted in drinking water was daily administered *via* oral gavage for 2 weeks previous 6-OHDA injection and followed for further 3 weeks (as indicated in “Material and Methods” section and in the timeline Figure 7A, total 5 weeks). Oral administration of the probiotic formulation did not generate neither mortality nor significant variances in the average body weights, in control and treated mice (Figure 7B). In Figure 7B also behavioral tests are shown. Notably, the cylinder test, considered to evaluate locomotor asymmetry in rodent models of CNS diseases, showed that striatum lesion induced a robust and significative decrease in mice motor performance, in particular, it was possible to appreciate a decrease of the use of contralateral paw. Interestingly, SLAB51 was able to counteract behavioral impairment induced by 6-OHDA inoculation, restoring the control conditions.

The Elevated Body Swing Test (EBST), index of asymmetrical motor performance of hemi-parkinsonian models in a drug-free state, in the same figure is shown. 6-OHDA-lesioned mice showed right-biased swings of 70% or greater respect to control animals, while probiotic formulation treatment was able to counteract this effect, in fact, at 2 weeks the animals showed the same behavior of SHAM group. These findings were confirmed by apomorphine test, a tool to monitor motor impairment and functional improvement [31]. In particular, the dopamine (DA) receptor agonist apomorphine (APO), acting post-synaptically and hyperstimulating supersensitive DA receptors in the denervated *striatum*, leads the animal to rotate in the opposite contralateral direction, i.e., away from the lesioned side. Indeed, in our experimental conditions, upon 6-OHDA mice showed increased contralateral rotations, while the 6-OHDA mice treated with the probiotic formulation had a similar behavior to SHAM groups (Figure 7).

**Figure 8:**
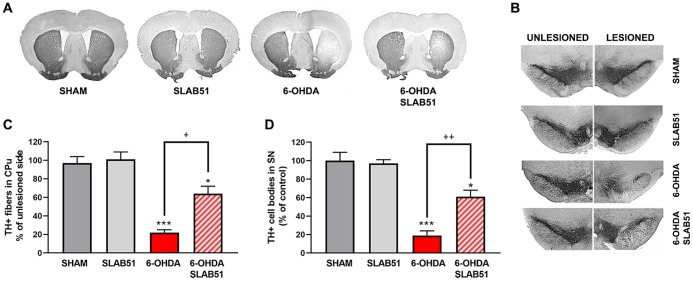
**Immunostaining of TH in dopaminergic neurons.** Transverse section taken through the substantia nigra pars compacta (SNc) and the ventral tegmental area (VTA), immunostained for TH to evaluate the dopaminergic-induced injury by stereotaxic injection of 6-OHDA in the right side. Histograms shows the percentage of TH+ fibers loss in striatum (CPu) and TH+ cell bodies in substantia nigra (SN) (expressed in percentage of unlesioned side). * p< 0.05, *** p< 0.0005 vs Ctr; + p< 0.05, ++ p< 0.005 vs 6-OHDA.

**Figure 9:**
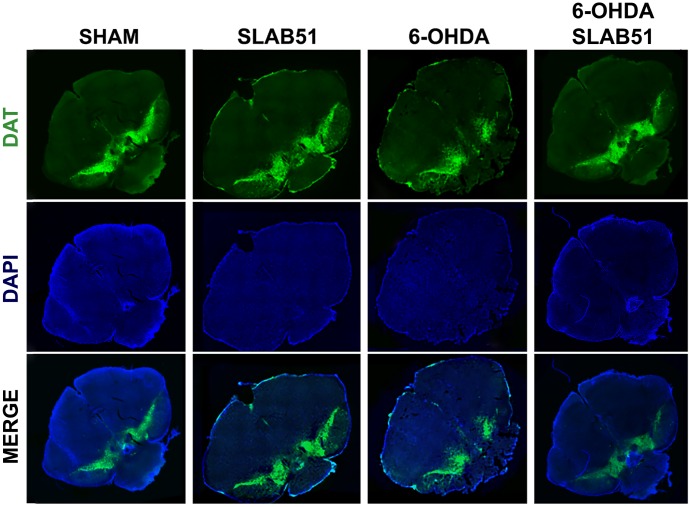
**Immunofluorescence for DAT in mice Substantia nigra.** Images were taken at confocal microscope at 20x magnification.

Further, the immunostaining of TH in dopaminergic neurons was performed; it was possible to observe in 6-OHDA-treated animal a marked decrease of TH immunoreactivity, while SLAB51 rescued dopaminergic neurons in both *substantia nigra* and *striatum* (CPu) (Figure 8). To further validate these data, immunofluorescence analyses for Dopaminergic Transporter (DAT) in mice *substantia nigra* were performed. As reported in Figure 9, a massive reduction of DAT fluorescence intensity was observed upon 6-OHDA inoculation (in the right side), while the probiotic formulation counteracted this effect, thus suggesting that SLAB51 exerted neuroprotective activities.

**Figure 10:**
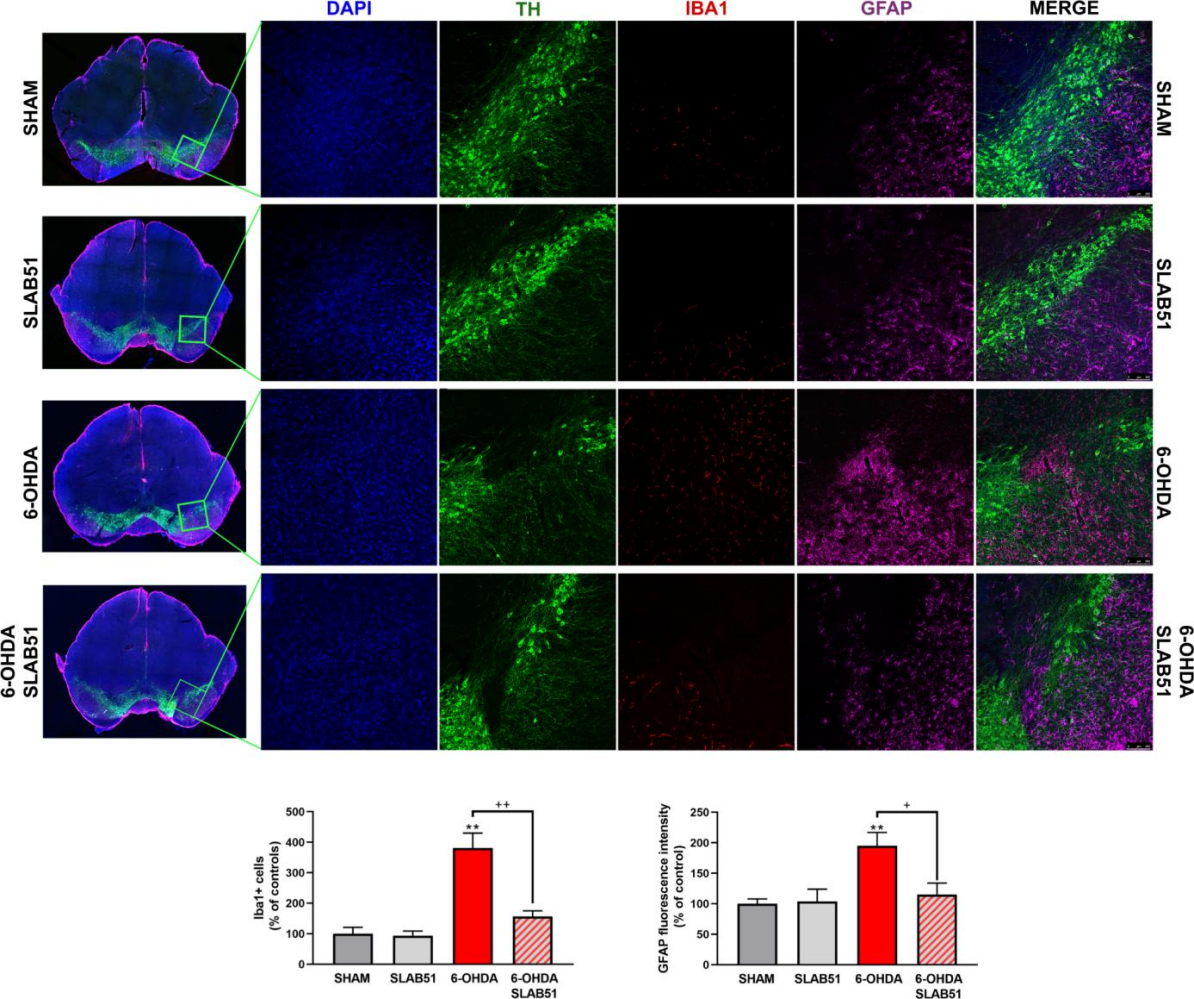
**Triple immunostaining at 20x magnification for Iba1, TH and GFAP, nuclei were counterstained with DAPI.** On the left it is possible to appreciate mosaic figures, while on the right inset at higher magnification for TH, Iba1 and GFAP staining and merge figures were reported. Histograms for Iba1 show the number of Iba1 + cells, while for GFAP the fluorescence intensity, as % of controls, is plotted. ** p< 0.005 vs Ctr; + p< 0.05, ++ p< 0.005 vs 6-OHDA.

Recent studies have demonstrated neuroinflammation and microglia activation in PD [30]. For this reason, immunofluorescence analyses and quantification for the specific marker of microglial activation, microglia ionized calcium-binding adapter molecule 1 (Iba1), and for astrogliosis, glial fibrillary acid protein (GFAP), in brain slices were performed (Figure 10). It is possible to observe a significant increase of Iba1 and GFAP fluorescence intensity in 6-OHDA slices, while SLAB51 was able to counteract 6-OHDA-induced effects, thus indicating a reduction in neuroinflammation.

**Figure 11:**
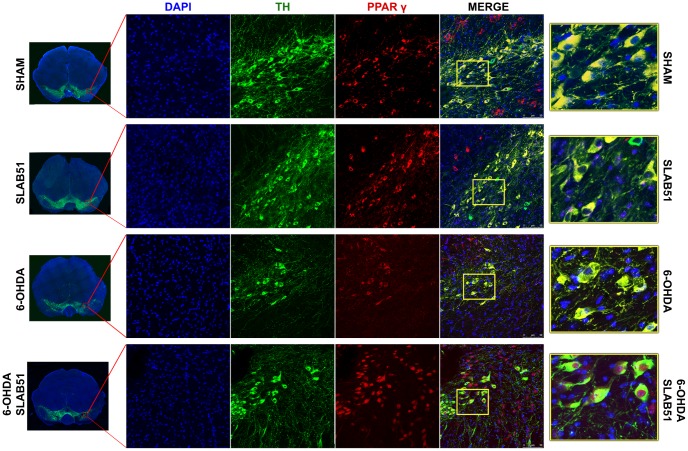
**Immunofluorescence analysis for PPARγ in substantia nigra.** On the left, the mosaic images obtained using confocal microscopy at 20x magnification were shown. In the center, double immunostaining at 40x magnification with TH and PPARγ as well as the merge figures were reported. On the right it is possible to observe the inset of the indicated boxes.

**Figure 12:**
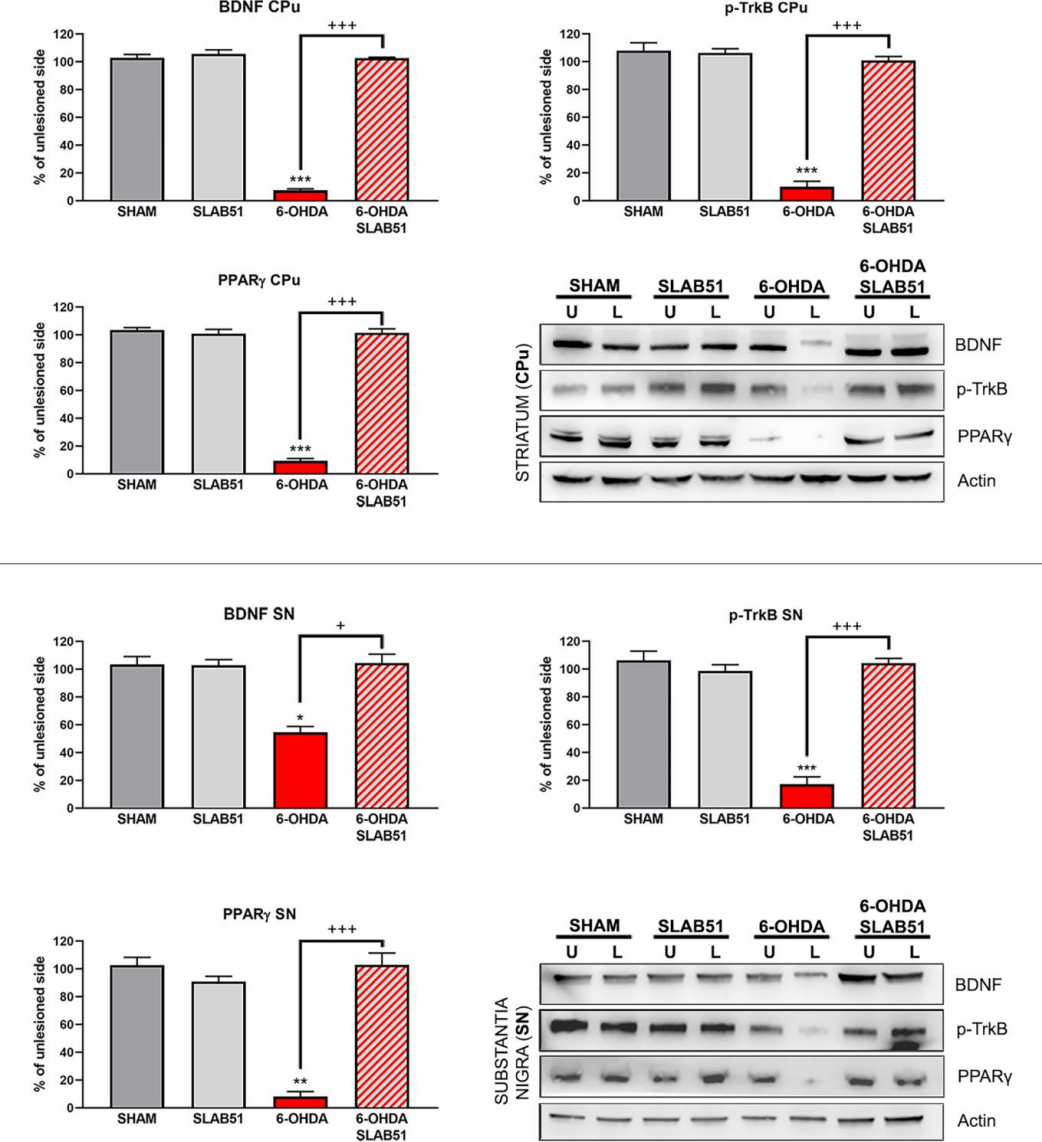
**Western blotting and relative densitometric analysis for mBDNF, p-TrkB and PPARγ in substantia nigra (SN) and striatum (CPu).** Results are mean ± SE of 3 experiments (n=3). * p< 0.005, ** p< 0.005, *** p< 0.0005 vs Ctr; + p< 0.005, +++ p< 0.0005 vs 6-OHDA. Representative WB images are shown.

On this basis, PPARγ, a ligand-dependent transcription factor involved in neuroinflammation, oxidative stress and energetic metabolism, that is also able to stimulate neurotrophins release (including BDNF) [30], was analyzed by immunofluorescence in brain slices (Figure 11). Interestingly, SLAB51-treated samples showed the transcription factor at nuclear level, while in 6-OHDA-treated animals, the fluorescence intensity for PPARγ was strongly decreased and, further, localized at cytoplasmic level. Western blotting analyses for PPARγ (Figure 12) demonstrated a restoration of this protein levels in 6-OHDA/SLAB51 treated animals to those of control group. Western blotting results, concomitant with PPARγ localized into the nucleus, may suggest that its activation could be responsible for the reduced neuroinflammation and for the neuroprotection *via* BDNF pathway, as confirmed by Western Blot analysis performed on *in vivo* samples. Indeed, BDNF and its receptor TrkB showed the same behavior of PPARγ, suggesting that SLAB51 was able to counteract the toxin-induced lesion both in *substantia nigra* and *striatum* (Figure 12).

**Figure 13:**
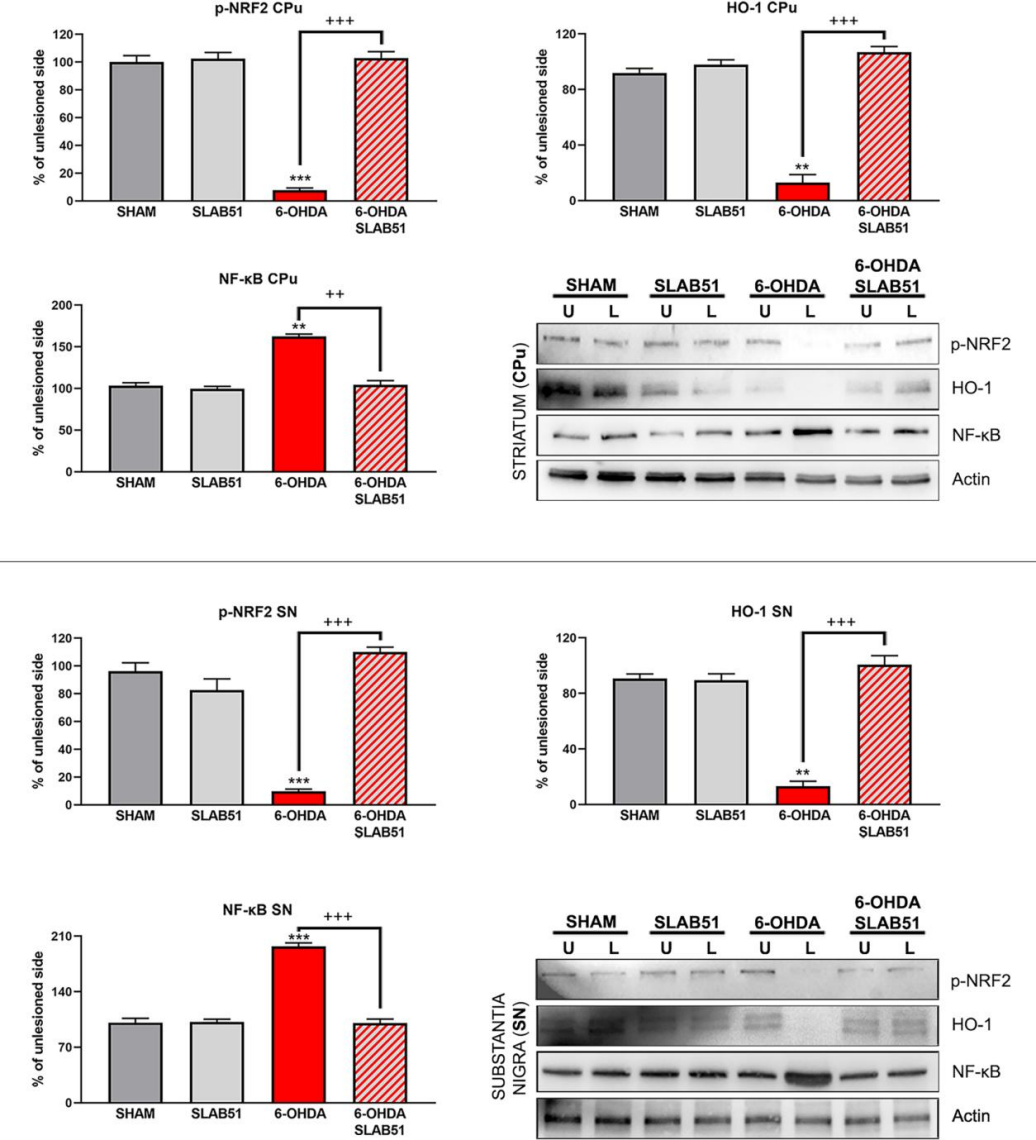
**Western blotting and relative densitometric analysis for p-Nfr2, HO-1 and NF-KB in SN and CPu.** Results are mean ± SE of 3 experiments (n=3). ** p< 0.005, *** p< 0.0005 vs Ctr; ++ p< 0.0005, +++ p< 0.0005 vs 6-OHDA. Representative WB images are shown.

Recent findings indicated that hemeoxygenase-1 (HO-1) is regulated by upstream regulators of PPARγ [32]. The antioxidant enzyme HO-1 with established cytoprotective effects has been demonstrated to modulate several pathological processes, including PD. Notably, HO-1 is involved in the release of neurotrophic factors, in the sustainment of dopaminergic neuronal survival in *substantia nigra*, and in preventing α-synuclein aggregation [33]. Interestingly, in our experimental conditions, 6-OHDA significantly decreased HO-1, while the probiotic formulation was able to counteract 6-OHDA effects, reverting the levels of HO-1 to those of control condition as shown in Figure 13.

In addition, nuclear transcription factor-erythroid 2 related factor (Nrf2) is able to bind the antioxidant response element (ARE) present in the HO-1 promoter region [34]. Nrf2 activity decreases with aging and represent one of the main PD risk factors, effectively, the increase of Nrf2 provides protection to dopaminergic neurons by counteracting oxidative stress injury [35]. As shown in Figure 13, p-Nrf2 (transcriptionally active) protein levels were significantly downregulated in 6-OHDA-injured animals, while SLAB51 was able to counteract 6-OHDA effects, both in *substantia nigra* and *striatum*.

Further, NRF2-ARE system interacts with nuclear factor kappa-light-chain-enhancer of activated B cells (NF-ĸB), a protein complex involved in cell survival and cytokine release, related to neurodegenerative and neuroinflammatory conditions. In fact, in our experimental conditions, NF-ĸB protein levels were upregulated upon 6-OHDA challenge, while the probiotic treatment reverted this protein to control conditions, both in *substantia nigra* and *striatum* (Figure 13), thus suggesting a control in neuronal inflammation and the immune response.

**Figure 14:**
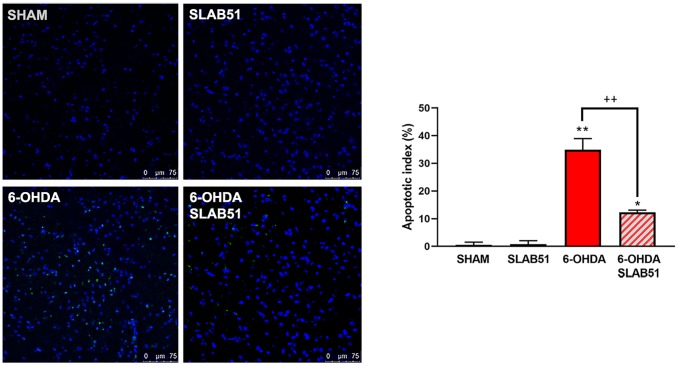
**TUNEL assays in mice substantia nigra. Figures were taken at confocal microscope at 40x magnification.** The graph shows apoptotic index obtained by counting positive nuclei. * p< 0.005, ** p< 0.005 vs ctr; ++< 0.005 vs 6-OHDA. Representative figures are reported.

Finally, to confirm the potential pro-survival effect of SLAB51, the apoptosis promotion in dopaminergic neurons of *substantia nigra* was analyzed by TUNEL assay. As reported in Figure 14, apoptosis dramatically increased in 6-OHDA treatment, while the presence of the probiotic mixture reduced apoptotic nuclei index (to about 10%), thus suggesting that the probiotic formulation protects against 6-OHDA-mediated apoptosis.

## DISCUSSION AND CONCLUSION

PD is a common neurodegenerative disorder, characterized by motor and non-motor symptoms, including abnormalities in the gut function, which may appear before the motor sign [13]. From a molecular point of view, PD underlying mechanisms include increased oxidative stress and inflammation [1]. To date, the available treatments can help to relieve PD-associated symptoms, but there is no cure to control the onset and the progression of this disorder.

A growing body of evidence reported that the use of probiotics can have positive influences on CNS disease, altering the gut microbiota, through the gut-brain axis [36], mediating numerous pathways such as immune, neural, inflammatory, and hormonal signaling [37].

Probiotics are live microorganisms residing in the intestine and are beneficial for their hosts and avoid certain diseases [38]. Recently, it has been reported that modulating the gut microbiota by using SLAB51, a mixture of bifidobacteria and lactobacilli, may delay Alzheimer’s disease progression by affecting different neuronal pathways [28].

On this basis, the aim of our study was to investigate the effects of SLAB51 formulation in PD. The effect of this formulation in 6-OHDA were first studied in the *in vitro* model.

In neurodegenerative diseases, including PD, reduced neurotrophic support has been reported [6, 39]. BDNF, a member of the neurotrophin family, maintains the survival and the differentiation of dopaminergic neurons. *In vitro*, BDNF counteracted the dopaminergic neurons death, indicating its potential use in the advance of neuroprotective therapeutic approaches for PD [40]. Accordingly, our *in vitro* studies indicated that the probiotic formulation SLAB51 modulates the BNDF pathway, increasing neuroprotective protein levels and decreasing the neuronal death proteins, confirming a neuroprotective effect exerted by the probiotics.

Thus, we investigated the probiotic *in vivo* assessing behavioral tests and, interestingly, SLAB51 was able to counteract the detrimental effect of 6-OHDA. The behavioral benefits using a therapeutic approach can be achieved through direct stimulation of the dopaminergic receptor or by protecting the dopaminergic neurons from 6-OHDA toxicity. Basing on the immunohistochemistry results, we can propose an involvement of a direct protection against dopaminergic neurons loss in the *substantia nigra*, thus protecting nigro-striatal pathway.

Besides the anti-inflammatory activity of SLAB51, detected by immunofluorescence, the antioxidant activity of the probiotic may also contribute to its neuroprotective effects *in vivo,* as suggested by the decrease of lipid peroxidation. Nrf-2 is a transcription factor involved in PD pathogenesis controlling cellular redox status via endogenous antioxidant systems, with concomitant anti-inflammatory effects. HO-1 is a Nrf2 target gene, which is at the core of Nrf2-mediated NFκB inhibition [41]. Indeed, in our *in vivo* experiments, the probiotic formulation tested was able to counteract 6-OHDA-induced dysfunction, reestablishing the activity of Nrf2/HO-1 pathway and inhibiting NFκB. In agreement, in a recent work on a progressive neurodegeneration mouse model induced by lipopolysaccharide, the treatment with a known PPARγ agonist, pioglitazone, was able to increase Nfr2 and HO-1, while reducing NFκB [33], thus supporting the pivotal role of activated-PPARγ in counteracting oxidative stress as well as neuroinflammation.

It is worth noting that the two models utilized in this research are quite different. In particular, the *in vivo* is a model that most resembles the clinical conditions, while the *in vitro* model is an isolated model, useful to dissect what happens in a single dopaminergic neuron. It is intriguing that, however, we obtained almost the same results in both models. It is possible to speculate that the common denominator may be the PPARγ that both *in vivo* and *in vitro* might be activated by some products or derivatives of bacteria metabolism. In this way, activated PPARγ may trigger anti-inflammatory and antioxidant activities as well as the increase in BDNF and its pro-survival pathways.

Our *in vivo* study showed that the probiotic administration was able to protect dopaminergic neurons and to improve behavioral impairments. This novel probiotic formulation was able also to counteract neuroinflammation and oxidative stress, characteristics of PD, reverting some underlying molecular pathways to control conditions, both *in vivo* and *in vitro*.

Overall, our findings propose SLAB51 as a promising candidate for PD prevention or treatment or as coadjuvant therapy, confirming that the modulation of the gut microbiota affects different pro-survival pathways, possibly leading to a delay of PD progression. Further studies will be necessary to analyze the microbiota composition of PD group *versus* probiotic-treated group, through amplification sequencing methods or hybridization on microarrays.

## MATERIALS AND METHODS

### *In vitro* experiments

### Preparation of SLAB51 bacterial lysates

Amount of 1 g of SLAB51 formulation (sold as Sivomixx, Mendes, Switzerland) has been suspended in 10 ml of Phosphate Buffer Saline (PBS, Euroclone, UK) for bacterial lysates preparation, subjected to centrifuge and sonication processes and at the final step the lysates were also filtrated to remove whole bacteria remaining as previously described by [42].

### MTS assay

To test cell viability, Cell Titer Cell Proliferation kit according to manufacturer’s instructions were used (Promega Corporation Madison, USA). The index of viability, which is dependent on formazan generated, was evaluated using an ELISA reader, Infinite F200 (Tecan, Swiss). Test was performed in quadruplicate. The results were reported as absorbance at 492 nm.

### *In vitro* model

The human SH-SY5Y cell line has been purchased from ECACC and cultivated in Dulbecco’s minimum essential medium, completed with 10% heat-inactivated FBS and 1% penicillin/streptomycin (Corning, USA) at 37°C in 95% O_2_ and 5% CO_2_ incubator (Thermo, USA). For the PD *in vitro* model, the SH-SY5Y cell line was differentiated with all trans-retinoic acid (10mM) and 3 days with phorbol (80nM). At 6 DIV, cells were treated with SLAB51 0.1 mg/ml of extract for 2 hours and then added 6-OHDA (Sigma Aldrich, USA) (35μM) for 24 hours. All experiments were performed at 19^th^ passage and the cell culture were tested to *Mycoplasma* presence (Mycoplasma PCR, abm, USA).

### Immunofluorescence

After culturing the cells as described above, were fixed in 4% PFA in PBS for 15 min and permeabilized in CH_3_OH for 7 minutes at −20° C. Cells were incubated in 4% BSA (Sigma Aldrich, USA) for 30 minutes then with the subsequent primary overnight at 4° C: rabbit polyclonal anti-β-Tubulin III (1:1000 Abcam, Cambridge, UK), rabbit polyclonal anti-TH (1:200, Novus Biologicals, Centennial, USA), mouse monoclonal anti-GAP43 (1:200, Abcam, Cambridge, UK). After several washings, coverslips were incubated with secondary antibodies, goat anti-mouse or anti-goat IgG Alexafluor 488 or 633 or 546 (1:2000 Life Technologies, California, USA), for 1h at RT. After different washes, Vectashield mounting with DAPI (Vector Laboratories Burlingame, USA) were used. All the samples were observed using confocal laser microscope (Leica, Wetzlar, Germany).

### Western blotting

Control and treated cells were collected and lysated as previously described and the protein amount were evaluated [43, 44]. 30 μg of proteins were loaded and separated on precast 4–20% gradient Bis-Tris gel in running buffer at 100 mV for 70 min followed by transfer to PVDF membranes using a semi-dry device (Thermo scientific, UK), then blocked in 5% no-fat milk for 30 minutes. Membranes were incubated with the subsequent primary antibodies overnight: anti-p-AKT (1:1000), anti-PI3K (1:1000 Cell Signaling, USA), anti p-CREB (1:500 Cell Signaling, USA), anti-pTrkB (1:2000 Cell Signaling, USA), anti-mBDNF (1:500 Abcam, UK), anti-pJNK (1:1000 Santa Cruz, USA), anti-p75(1:1000 Abcam, UK), anti-pro-BDNF(1:1000 Millipore, USA), anti-pERK5 (1:1000 Cell Signaling, USA), anti p-ERK1,2 (1:1000 Santa Cruz, USA). After different washes, membranes were incubated with 1:10000 horseradish peroxidase-conjugated anti-rabbit IgG or anti-mouse IgG. The protein bands were detected, normalized and analyzed to actin (housekeeping). Anti-β-actin (HRP-conjugate) (1:10000) has been used. To reprobe, membranes have been stripped with Restore stripping buffer (Thermo Scientific, UK) following manufacturer’s instructions.

### *In vivo* experiments

### Animals

Animal handling and surgical procedures were performed in order to minimize discomfort and pain, according to the ethical regulations of the European Communities Council (Directive 2010/63/EU, prot #542/2019-PR).

For 6-OHDA lesion experiments, male C57BL/6 mice purchased from Charles River (Massachusetts, USA) 9-week-old were used. Animals (n=30) were kept in ventilated cages (Tecniplast, Germany) under a 12-hour light/12-hour dark cycle with water and food *ad libitum*. Stereotaxic (Stoelthing, USA) injections of 6-OHDA were performed as previously reported [45]. Briefly, mice were subjected to anesthesia (xylazine (10 mg/kg) and ketamine (200 mg/kg) and then 4 μg of 6-OHDA containing 0.2% L-ascorbic acid (Sigma Aldrich, USA) in saline solution or saline solution with 0.2% L-ascorbic acid (SHAM group) were injected into the right region of the striatum (coordinates relative to bregma: medial-lateral +0.18 cm; anteroposterior + 0.04 cm; dorsal-ventral +0.35 cm) with a rate of 0.5 μl/minute using single syringe nano Infusion KDS 310 (KD Scientific, USA). After injection, we waited for 5 minutes before removal.

### Treatment

SLAB51 formulation was provided by Mendes Sa (Switzerland). SLAB51 was freshly prepared dissolving one sachet (1,5g/200 billion of bacteria) in 10 ml of drinking water and the treated mice received 270 μl using oral gavage (corresponding at around 5,4 billion, based on a weight human/weight mice ratio). SLAB51 was administered daily for 2 weeks previous 6-OHDA injection and followed for further 3 weeks. Control group received SLAB51 only.

### Behavioral tests

### Elevated body swing test

All investigators performing the behavioral tests were blinded to the treatment condition. To perform EBST, mice were gently picked up at the base of the tail and the direction of the swing, either left or right, was considered until 20 swings as described by [46].

### Cylinder test

Cylinder rearing test [47] was adjusted for use in mice to evaluate forelimb use during normal exploratory behavior and was conducted before 6-OHDA lesion and 1, 2, 3 weeks after the first lesion. Each mouse was positioned in a Plexiglass cylinder 25 cm in height and 11.48 cm in diameter. Spontaneous forelimb contacts were recorded until 20 contacts for each animal (2 not consecutive times). The number of paired and impaired forelimb contacts were evaluated as percentage of total contacts observed in the entire observation time.

### Morphological analysis

Animals were deeply anesthetized with ketamine/ xylazine, before being sacrificed by transcardial perfusion. Mice were perfused at RT with phosphate buffer saline (PBS), followed by 4% PFA in 0.12M phosphate buffer, pH 7.6. The brains were placed overnight in 4% PFA, then cryoprotected in 30% sucrose solution in 0.1M phosphate buffer (PB). Brains from each mouse were embedded in the OCT (Sigma Aldrich, Saint Louis, USA). The blocks were cut by a cryostat to obtain coronal 40 μm thick sections following “Paxinos and Franklin's the Mouse Brain in Stereotaxic Coordinates” (Elsevier).

### Immunohistochemistry

Free floating sections were incubated in a 0.3% hydrogen peroxide solution, for 10 min, protected from the light, to block internal peroxidases, and then in PBS 0.5% Triton X-100, 4% BSA for 1 h, RT. Sections were then incubated overnight at 4°C with rabbit polyclonal anti-TH (1:500), in PBS containing 0.4% Triton X-100. In control sections, the primary antibody was omitted. After incubation for 2h at RT with goat anti-rabbit IgG-HRP (Sigma, B7401), 1:100 in PBS containing 0.4% Triton X100, immuno-complexes were revealed using 3,3′-diamino-benzidine (DAB Substrate Kit for Peroxidase, Vector) as the chromogen. After extensive washing, sections were dehydrated and mounted with Permount (Fisher Scientific, US). Slides were observed with a Leica S8 Apo microscope equipped with EC3 camera. To quantify TH+ cells, 3 slices were used *per* each group.

### Immunofluorescence

For immunofluorescence experiments, sections were processed as reported in “immunohistochemistry” section and incubated for 24 hours at 4°C with the subsequent primary antibodies: rabbit polyclonal anti-TH (1:500), anti-NeuN (1:1000), anti DAT (1:1000), anti-Iba1 (1:500), anti-GFAP (1:500). Sections were rinsed with PBS and then incubated for 2h at RT in BSA containing 0.4 % Triton X-100, and secondary antibodies Alexa488 conjugated donkey anti-rabbit IgG 1:500 or Alexa633 conjugated donkey anti-mouse IgG 1:500 and Alexa596 conjugated anti- chicken IgG 1:500 (Life Technologies, California, USA). Controls were performed by omitting the primary antibody. Image acquisition in a Leica TCS SP5 confocal microscope was performed and then analyzed by ImageJ software. In particular, the average number of Iba1 + cells per section was revealed in five regularly spaced sections of the SN *per* animal. To quantify GFAP intensity fluorescence, ImageJ analysis software was used and reported in the graph as fluorescence intensity/% of control, analyzing 9 different fields for each condition (*n* = 3 mice each group; 3 fields *per* mouse).

The representative mosaic images provided were subjected to rotation in order to get all the figures orientated at the same direction.

### Western blotting

Under stereomicroscope, *substantia nigra* and *striatum* were isolated and the different regions were freshly lysate using pestles, protein extracted were dosed as previously described [43]. Tissues lysates containing 10μg of protein were separated on 4–13% gradient Bis-Tris gel in running buffer at 100 mV for 80 min. Proteins were transferred into PVDF membranes using a semi-dry device (Thermo scientific, UK). Membranes were washed in tris-buffered saline with 0.05% Tween20, and blocked in 5% no-fat milk for 1 h at RT. Membranes were then incubated overnight at 4°C with the following primary antibodies, diluted in the same blocking solution: anti p-NRF2 (1:5000 Abcam, UK), anti-NFKB (1:2000 Abcam, UK) anti-p-TRKB (1:2000 Cell Signaling), anti-BDNF (1:500 Abcam, UK), anti-PPARγ (1:500, Thermo, USA) anti-HO1(1:1000 Santa Cruz, USA) at 4°C overnight and then incubated with 1:10000 HRP-conjugated anti-rabbit IgG or anti-mouse IgG. Protein bands were detected with West Pico luminol (Thermo scientific) following kit’s datasheet. Through Alliance Q9 (Uvitec, Cambridge, UK) image chemiluminescent bands were detected and using ImageJ program we analyzed each band intensity normalized as indicated in the “Wester Blotting” *in vitro* section.

### TUNEL assay

The *substantia nigra* (SN*)* region of the mouse brain was cut at 40 μm on a cryostat and stored at −80 °C. To perform terminal transferase-mediated dUTP nick end-labeling (TUNEL) analyses, sections were fixed in 4% PFA for 30minutes and then washed several times with room temperature PBS. Then, the sections were incubated in cold ethanol/acetic acid 2:1 for 5mins and washed in PBS again. The labeling of neuronal apoptosis in SN sections was performed using the apoptosis detection kit purchased from ThermoScientific (USA), which is based on the *in situ* TUNEL technique using terminal deoxynucleotidyl transferase (TdT) and the images though confocal microscope were acquired (Leica TCS SP5).

### Statistical analysis

Statistical analysis has been performed by t-test, using PRISM 8 software. For statistical studies, *P<0.05 has been set as statistically significant.
